# Lineage-specific gene radiations underlie the evolution of novel betalain pigmentation in Caryophyllales

**DOI:** 10.1111/nph.13441

**Published:** 2015-05-13

**Authors:** Samuel F Brockington, Ya Yang, Fernando Gandia-Herrero, Sarah Covshoff, Julian M Hibberd, Rowan F Sage, Gane K S Wong, Michael J Moore, Stephen A Smith

**Affiliations:** 1 Department of Plant Sciences, University of CambridgeCambridge, CB2 3EA, UK; 2 Department of Ecology & Evolutionary Biology, University of Michigan830 North University Avenue, Ann Arbor, MI, 48109-1048, USA; 3 Departamento de Bioquímica y Biología Molecular A, Unidad Docente de Biología, Facultad de Veterinaria, Regional Campus of International Excellence ‘Campus Mare Nostrum’, Universidad de MurciaE-30100, Espinardo, Murcia, Spain; 4 Department of Ecology and Evolutionary Biology, University of Toronto25 Willcocks Street, Toronto, ON, M5S 3B2, Canada; 5 Department of Biological Sciences, University of AlbertaEdmonton, AB, T6G 2E9, Canada; 6 Department of Medicine, University of AlbertaEdmonton, AB, T6G 2E1, Canada; 7 BGI-Shenzhen, Beishan Industrial ZoneYantian District, Shenzhen, 518083, China; 8 Department of Biology, Oberlin College119 Woodland St, Oberlin, OH, 44074-1097, USA

**Keywords:** anthocyanin, betalains, Caryophyllales, lineage-specific genes, pigmentation, taxonomically restricted genes

## Abstract

- Betalain pigments are unique to the Caryophyllales and structurally and biosynthetically distinct from anthocyanins. Two key enzymes within the betalain synthesis pathway have been identified: 4,5-dioxygenase (DODA) that catalyzes the formation of betalamic acid and CYP76AD1, a cytochrome P450 gene that catalyzes the formation of cyclo-DOPA.
- We performed phylogenetic analyses to reveal the evolutionary history of the DODA and CYP76AD1 lineages and in the context of an ancestral reconstruction of pigment states we explored the evolution of these genes in relation to the complex evolution of pigments in Caryophylalles.
- Duplications within the CYP76AD1 and DODA lineages arose just before the origin of betalain pigmentation in the core Caryophyllales. The duplications gave rise to DODA-α and CYP76AD1-α isoforms that appear specific to betalain synthesis. Both betalain-specific isoforms were then lost or downregulated in the anthocyanic Molluginaceae and Caryophyllaceae.
- Our findings suggest a single origin of the betalain synthesis pathway, with neofunctionalization following gene duplications in the CYP76AD1 and DODA lineages. Loss of DODA-α and CYP76AD1-α in anthocyanic taxa suggests that betalain pigmentation has been lost twice in Caryophyllales, and exclusion of betalain pigments from anthocyanic taxa is mediated through gene loss or downregulation. [Correction added after online publication 13 May 2015: in the last two paragraphs of the Summary the gene name CYP761A was changed to CYP76AD1.]

## Introduction

Comparative genomic analysis has revealed a significant fraction of genes that occur only in defined organismal lineages, and which have been variously termed orphan genes, taxonomically restricted or lineage-specific genes (Khalturin *et al*., 2009). Several studies have demonstrated that lineage-specific gene radiations can contribute to unique evolutionary changes and novel phenotypic adaptation (Khalturin *et al*., 2008; Fry *et al*., 2010; Simola *et al*., 2013). In a recent phylotranscriptomic analysis of the flowering plant order Caryophyllales sensu APG III (Bremer *et al*., 2009) we identified gene lineages that exhibit substantially higher rates of gene duplication than in non-Caryophyllales outgroups (Yang *et al*., 2015). The Caryophyllales are well recognized for extraordinary levels of morphological and physiological adaptation, and in this context, we proposed that these clade-specific, highly duplicated gene lineages might contain important novel genes associated with unusual evolutionary adaptations. Consistent with this hypothesis, we found that two of the most highly duplicated gene lineages among Caryophyllales transcriptomes encode a cytochrome P450 gene and a 4,5-dioxygenase gene, respectively (Yang *et al*., 2015), that had previously been functionally implicated in the synthesis of betalains, a group of pigments unique to core Caryophyllales.

**Fig. 1:**
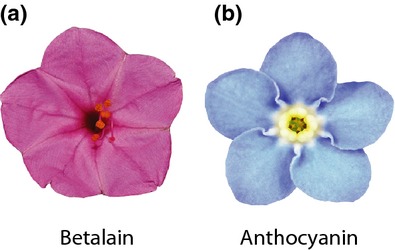
(a) Pink hue of a betalain-pigmented flower (*Mirabilis jalapa*). (b) Blue anthocyanins, a color that is not obtainable with betalains (*Myosotis* sp.).

In the core Caryophyllales, betalains can largely substitute for the otherwise ubiquitous anthocyanins (Bischoff, 1876; Mabry, 1964), which are the dominant form of pigmentation across land plants (Campanella *et al*., 2014) (see Fig. 1). Betalains are water-soluble, possess high antioxidant and free radical scavenging activities (Escribano *et al*., 1998; Cai *et al*., 2003; Wu *et al*., 2006), exhibit preventative properties with respect to several types of cancer (Lu *et al*., 2009; Khan *et al*., 2012; Krajka-Kuźniak *et al*., 2012). Given the clear health benefits of betalains (Gandía-Herrero *et al*., 2013), there is interest in expressing the betalain synthesis pathway in the anthocyanic background of food crops (Harris *et al*., 2012; Hatlestad *et al*., 2012). Understanding the restricted distribution of betalains within Caryophyllales may therefore have implications for human health and nutrition (Gandía-Herrero *et al*., 2013). Although some of the genetic components necessary for betalain pigmentation are suggested to be present in anthocyanic plants outside of the Caryophyllales, thereby minimizing the steps needed to express the pathway in heterologous species, other elements appear to be Caryophyllales-specific (Harris *et al*., 2012). In this context, a clear understanding of the origin and evolution of the genetic components comprising the betalain synthesis pathway is valuable.

**Fig. 2:**
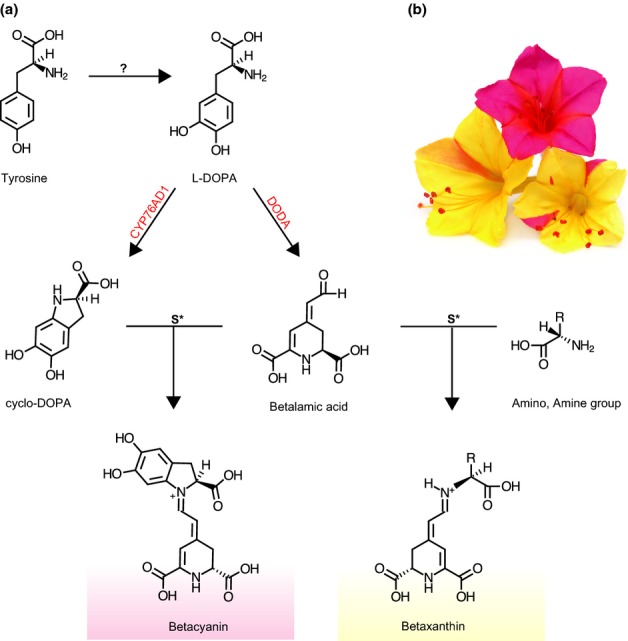
(a) Outline of the betalain biosynthetic pathway with the key enzymes CYP76AD1 and 4,5-dioxygenase (DODA) marked in red. CYP76AD1 catalyses the conversion of l-3,4-dihydroxyphenylalanine (l-DOPA) into cyclo-DOPA, whereas DODA catalyzes the conversion of l-DOPA to betalamic acid. Betalamic acid spontaneously cyclizes with cyclo-DOPA to give red-pigmented betanidins, whereas betalamic acid can conjugate with amino and amine groups to generate yellow betaxanthins. (b) Variegated forms of *Mirabilis jalapa*, with yellow forms in which betaxanthins are the dominant pigment and pink forms in which betacyanins are dominant.

Betalains are structurally and biosynthetically distinct from the more common anthocyanin pigments, with the former derived from tyrosine, and the latter from phenylalanine (Clement & Mabry, 1996). Synthesis of betalains is proposed to require three enzyme-mediated steps (Hatlestad *et al*., 2012) (see Fig. 2). The enzyme or enzymes responsible for the first step are currently unknown but are possibly tyrosinase-like in action (Christinet *et al*., 2004), converting tyrosine to l-3,4-dihydroxyphenylalanine (l-DOPA). l-DOPA is then a key substrate in the formation of 4,5-seco-DOPA, which spontaneously cyclizes to betalamic acid (Christinet *et al*., 2004), and is also a substrate in the formation of cyclo-DOPA (Tanaka *et al*., 2008). Betalamic acid and cyclo-DOPA in turn spontaneously condense to form red betanidin pigments, whereas betalamic acid can condense with additional amine- or amino-groups to form yellow betaxanthin pigments. The genes contained within our highly duplicated gene lineages encode proteins that catalyze two of these key steps: (1) 4,5-dioxygenase (DODA) catalyzes the formation of betalamic acid (Christinet *et al*., 2004; Sasaki *et al*., 2009; Gandía-Herrero & García-Carmona, 2012); and (2) CYP76AD1, a cytochrome P450 gene, catalyzes the formation of cyclo-DOPA (Hatlestad *et al*., 2012).

In the betalain-producing species of the Caryophyllales, anthocyanins have never been detected (Bate-Smith & Lerner, 1954; Mabry, 1964) and, conversely, the anthocyanic lineages within Caryophyllales are not known to produce betalains (Clement & Mabry, 1996). On the basis of these data, it has been proposed that anthocyanins and betalains are mutually exclusive (Stafford, 1994; Clement & Mabry, 1996). Furthermore, the occurrence of anthocyanins and betalains exhibit interesting patterns of homoplasy such that anthocyanic lineages are either nested within (e.g. Molluginaceae s.s. and some of its recent segregates such as *Kewa* Kewaceae; previously included in *Hypertelis*; Christenhusz *et al*., 2014) or sister to (e.g. Caryophyllaceae) betalain-pigmented lineages (Brockington *et al*., 2011). Therefore, in understanding the evolution of betalain pigmentation, it is necessary to explain these three observations; that is, the unique origin of betalains, their apparent mutual exclusivity with anthocyanins, and the homoplastic distribution of the two pigment types (Brockington *et al*., 2011).

Previous analyses have determined that three key structural enzymes within the anthocyanin synthesis pathway – chalcone synthase (CHS), dihydroflavonol-4-reductase (DFR) and anthocyanin synthase (ANS) – are maintained in betalain-pigmented taxa, but in the case of ANS are downregulated except in seeds (Shimada *et al*., 2004, 2005). The maintenance of this structural pathway is thought to be due to the continued requirement of proanthocyanidins in seeds, and the lack of anthocyanins in other tissues in betalain-pigmented taxa is attributed to downregulation or suppression at the regulatory level (Shimada *et al*., 2004, 2005). By contrast, the evolutionary fate of betalain synthesis genes in anthocyanic Caryophyllales is undetermined, and little is known about the presence or absence of specific betalain synthesis genes in anthocyanic species outside of the Caryophyllales. Putative homologs of DODA have been isolated from a range of anthocyanic taxa across angiosperms (Christinet *et al*., 2004) but their precise relationship to betalain-specific isoforms is unknown. Similarly, the cytochrome P450 gene family that contains CYP76AD1 has radiated across the land plants (Mizutani & Ohta, 2010), but the evolutionary origin of CYP76AD1 within this large gene family is unknown.

Here, we explore the highly duplicated CYP76AD1 and DODA lineages in Caryophyllales, in order to understand the unique origin of the betalain synthesis pathway and its fate in anthocyanic taxa within Caryophyllales. We demonstrate that the earliest duplications in these gene lineages were apparently coincidental and gave rise to isoforms specific to betalain synthesis immediately before the earliest inferred origin of betalain pigmentation, and that these betalain-specific duplicate loci were then mostly lost or downregulated in the anthocyanic Molluginaceae and Caryophyllaceae. This pattern of duplication and loss is consistent with a single origin of betalain-specific function in core Caryophyllales. We further demonstrate that the betalain-specific isoforms of CYP76AD1 and DODA in *Beta vulgaris* are in close physical proximity on chromosome 2, suggesting the possibility of a metabolic operon and supporting the idea of their coincident duplication. We also analyze patterns of positive selection within and among these radiating DODA and CYP76AD1 lineages and implicate key residues in the neofunctionalization of the betalain-specific loci. Finally, we report the asymmetric diversification of the betalain-specific DODA genes that may be implicated in the evolution of color within the betalain pigment system.

## Materials and Methods

Genome and transcriptome data from a total of 100 species were included in our analysis, including both amino acid and coding sequence (see Supporting Information Table S1). Among them, 95 were ingroup species representing 26 of the 34 families in Caryophyllales (Bremer *et al*., 2009). Of these, two were from annotated genomes and the remaining 93 were from transcriptomes. Fifty-nine transcriptomes were obtained from the One Thousand Plants (1KP) Consortium, including 58 that were previously published (Finn *et al*., 2014; Matasci *et al*., 2014; Wickett *et al*., 2014) and one as yet unpublished (*Portulacca amilis*, Table S1). Raw reads for another 20 transcriptomes were downloaded from the NCBI Sequence Read Archive (SRA; Table S1). Fourteen transcriptomes were newly generated for this study (Table S2). RNA isolation was carried out using the Bio-Rad Aurum Total RNA Mini Kit (Bio-Rad Life Science Research, Hercules, CA, USA) following the manufacturer's instructions, or using TRIzol® Reagent (Life Technologies, Thermo Fisher Scientific, Waltham, MA, USA) followed by a DNase treatment using the TURBO DNA-free™ Kit (Life Technologies, Thermo Fisher Scientific). Total RNA was quantified on an Agilent 2100 Bioanalyzer (Agilent Technologies Inc., Santa Clara, CA, USA). Libraries were prepared using the TruSeq Stranded mRNA Sample Prep Kit (Illumina Inc., San Diego, CA, USA), and were quantified using an Agilent 2100 Bioanalyzer. Six or eight libraries were multiplexed per lane on an Illumina HiSeq2000 sequencer at the University of Michigan DNA Sequencing Core. Newly generated reads were deposited in SRA (BioProject: PRJNA280277).

All raw reads that were newly generated or downloaded from SRA were filtered following the same procedures as Yang *et al*. (2015). *De novo* assembly was carried out using Trinity v20140413p1 with default settings (Grabherr *et al*., 2011) for datasets from SRA, and with the stranded ‘RF’ setting for the newly generated sequences. All assembled transcripts were translated using TransDecoder v16JAN2014 (Haas *et al*., 2013) taking Pfam domain information into account. Amino acid and coding sequences of seven species were obtained from genome annotations in Phytozome v9 (Goodstein *et al*., 2012) or respective publications (The Arabidopsis Genome Initiative, 2000; Tomato Genome Consortium, 2012; Huang *et al*., 2013; Ibarra-Laclette *et al*., 2013; Dohm *et al*., 2014; Yagi *et al*., 2014).

Amino acid and mRNA sequences from the *Beta vulgaris DODA* gene, as well as from the *B. vulgaris CYP76AD1* gene and its orthologs in *Amaranthus cruentus* and *Mirabilis jalapa*, were obtained from Hatlestad *et al*. (2012). The *B. vulgaris DODA* and *CYP76AD1* amino acid sequences were used to search against each of the 101 amino acid datasets using SWIPE v2.0.9 (Rognes, 2011) with an *E*-value cutoff of 10. We used a high *E*-value cutoff to maximize the sensitivity of searches in order to identify short and incomplete sequences. Positive hits with at least 40% amino acid identity to the *B. vulgaris DODA* gene or the *B. vulgaris CYP76AD1* gene were included in downstream analyses, except for the search for *CYP76AD1* homologs in *Arabidopsis thaliana*, for which hits with at least 20% amino acid identity were included. All utilized DODA and CYP76AD1 homologs are listed in Tables S3 and S4, respectively. Sequences were deposited with GenBank; DODA sequences KR376141–KR376346, and CYP76AD1 sequences KR376350–KR376500.

In order to estimate the gene trees for *DODA* and *CYP76AD1*, an iterative set of alignment and phylogenetic estimation steps was conducted. An initial alignment was carried out for each of the two genes using MAFFT v7.154b using the default settings (Katoh & Standley, 2013), and low occupancy columns were trimmed using Phyutility v2.2.6 (-clean 0.01) (Smith & Dunn, 2008). A phylogeny was estimated with FastTree v2.1.7 (-wag) (Price *et al*., 2010) and the alignment was then refined using SATé v2.2.7 (–iter-limit = 3) (Liu *et al*., 2012). The resulting refined alignment was again trimmed (phyutility -clean 0.05) and a tree was then estimated again in FastTree (-wag). Branches longer than 1.5 were assumed to be due to distantly related paralogs and/or to assembly artifacts and were pruned. For *CYP76AD1*, which is part of a very large gene family, we extracted the clade of sequences containing the *B. vulgaris CYP76AD1* sequence together with the closest outgroup sequences for subsequent analysis. Sequences were realigned using PRANK v140110 (Löytynoja, 2014), poorly aligned sequences were removed from the alignment, trimmed (phyutility -clean 0.1), and the phylogeny was estimated by RAxML v8.0.0 (-m PROTCATWAG) (Stamatakis, 2014). Tips with branches longer than 1.0 were removed from the alignment, and the phylogeny re-estimated with 200 rapid bootstrap replicates. In addition to PRANK, we also used SATé and MAFFT for alignments and the resulting trees were highly similar. MAFFT and SATé tend to overalign (force regions to align even when they are highly divergent) whereas PRANK tends to do the opposite (introduce lots of gaps in highly divergent regions). For both DODA and CYP76AD1 lineages, the core Caryophyllales clade containing the known *B. vulgaris* gene was extracted and realigned, followed by removal of poorly aligned sequences and tree inference using RAxML with 100 bootstrap replicates. Analyses were repeated on the alignment using codon-aligned coding DNA sequence (CDS) data by RAxML and were found to be congruent with the amino acid-derived topologies. Phylogenies were rooted with non-Caryophyllales eudicot representatives of these orthogroups for which whole genome sequences could be obtained (e.g. *Solanum*, *Arabidopsis*).

All functionally characterized CYP761AD1 loci were retrieved from the literature and their location mapped onto the phylogeny, including: *MjCYP76AD3* (GenBank accession HQ656026) from *M. jalapa* (Hatlestad *et al*., 2012; Suzuki *et al*., 2014); *AcCYP76AD2* (HQ656025) from *A. cruentus* (Hatlestad *et al*., 2012); and *BvCYP76AD1* from *B. vulgaris* (Hatlestad *et al*., 2012). Similarly, all functionally characterized DODA loci were retrieved from the literature and mapped onto the phylogeny, including: *PgDODA* from *Portulaca grandiflora* (Christinet *et al*., 2004); *MjDODA* from *M. jalapa* (Sasaki *et al*., 2009); and *BvDODA* from *B. vulgaris* (Hatlestad *et al*., 2012). The loci from *B. vulgaris* belonging to the CYP76AD1 and DODA lineages were blasted against the genome of *B. vulgaris* (RefBeet-1.1) in order to reveal their relative genomic location (Dohm *et al*., 2014).

Codon models were used to test for the presence of positive selection during the evolutionary history of both the CYP76AD1 and DODA lineages. To avoid missing data, only sequences that were complete between codons 39 and 436 in CYP76AD1 (codons numbered on the basis of *B. vulgaris* CYP76AD1-α) and between 65 and 275 in DODA (codons numbered on the basis of *B. vulgaris* DODA-α) were included in analyses. Topologies were then re-estimated from the reduced alignment using RAxML (GTR + I + G). After confirming that the resulting tree topologies were congruent with trees derived from the original, larger alignments, they were subsequently used as reference topologies for the codon model analyses. CodeML as implemented in PAML v4.4 (Yang, 2007) was used to optimize codon models and estimate dN : dS values (ω) among branches of the RAxML-derived trees and at sites within the sequence alignments. CodeML estimation was performed using the clean data function that ignores all codons with ambiguous or missing data in the sequence alignment.

We employed branch-site model A (Yang & Nielsen, 2002) to test the occurrence of positive selection on individual codons along specific branch groups (model = 2 and NS sites = 2). Model A assumes that the branches in the phylogeny are divided *a priori* into foreground and background clades, where only the former may have experienced positive selection. For these analyses, all branches within the betalain-specific clades CYP76AD1-α and DODA-α were selected as the foreground and remaining branches of the topology outside of these clades served as background. Using CodeML, we analyzed the occurrence of positive selection along codon sites by comparing model M1a with model M2a (Yang, 2007). Codons evolving under positive selection in foreground branches were identified with a posterior probability > 0.95, as estimated by the Bayes Empirical Bayes procedure (Yang, 2007).

Ancestral state reconstruction analyses were performed in Mesquite (version 3.03, build 564) (Maddison *et al*., 2015) using a likelihood-based approach and an Mk1 model that allows forward and reverse rates to be the same (estimated rate 3.12701442, −log L.: 15.35774177). The tree topology was derived from a concatenated *matK*/*rbcL* dataset from Brockington *et al*. (2009) and Brockington *et al*. (2011), and was reanalyzed with GARLI 2.0 under a GTR + I + G model (Zwickl, 2000). Data on pigment content of the operational taxonomic units (OTUs) were obtained from a literature survey (primarily: Mabry, 1964; Clement & Mabry, 1996) and are the same as those contained in the dataset previously published by Brockington *et al*. (2011). OTUs were coded as anthocyanic, betalainic or missing data.

## Results

**Fig. 3:**
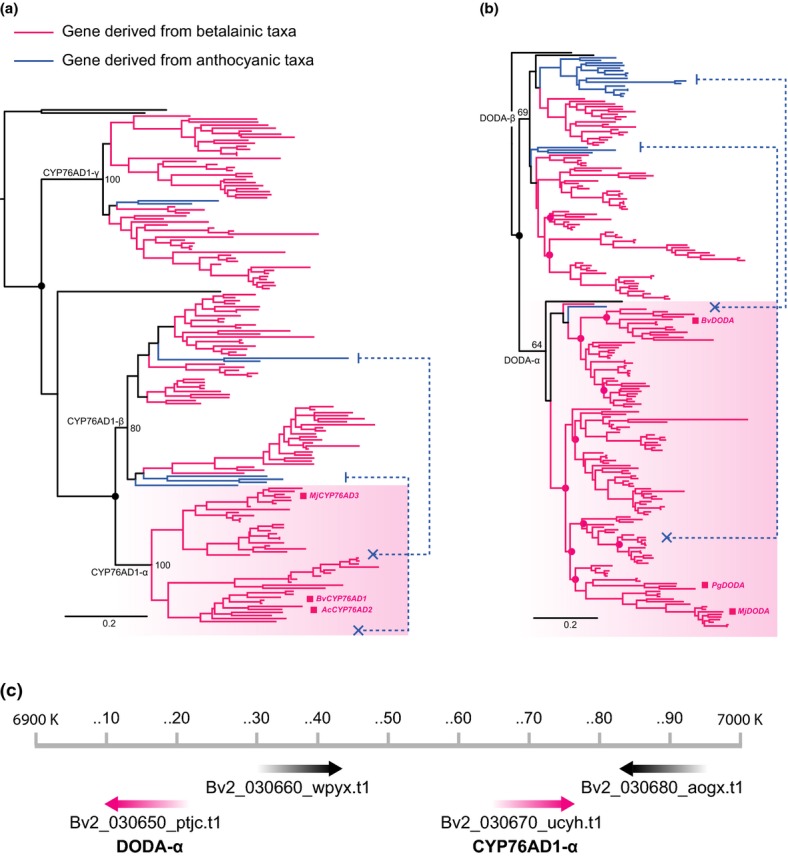
(a) Phylogeny of the CYP76AD1 lineage, delimiting the CYP76AD1-α, -β and -γ clades, indicating the branching patterns amongst the three clades and their bootstrap support values. Black lines are early diverging Caryophyllales lineages (Microteaceae or Physenaceae). Duplication events are marked with closed circles. CYP76AD1 homologs derived from betalain-producing taxa are marked pink and homologs derived from anthocyanic taxa are marked blue. Blue dashed lines indicate that no CYP76AD1-α orthologs were recovered from anthocyanic transcriptomes or genomes, in contrast to CYP76AD1-β and -γ. The phylogenetic locations of the three functionally characterized betalain-specific CYP76AD1 genes (*MjCYP76AD3*, *AcCYP76AD2*, and *BvCYP76AD1*) are marked within the CYP76AD1-α clade. (b) Phylogeny of the 4,5-dioxygenase (DODA) lineage, containing DODA-α and -β clades, and their bootstrap support values. Grey lines are outgroups. Duplication events are marked with closed circles. Blue dashed lines indicate that in contrast to DODA-β, almost no DODA-α orthologs were recovered from anthocyanic transcriptomes or genomes. In contrast to DODA-β, no DODA-α orthologs were recovered from anthocyanic transcriptomes or genomes. The phylogenetic locations of the three functionally characterized betalain-specific DODA orthologs (*MjDODA*, *PgDODA* and *BvDODA*) are marked within the DODA-α clade. (c) Map of the region of *Beta vulgaris* chromosome 2 that contains the two functionally related but nonhomologous genes DODA-α (Bv2_030650_ptjc.t1) and CYP76AD1-α (Bv2_030670_ucyh.t1).

The CYP76AD1 lineage underwent a minimum of three duplication events early on in the history of the core Caryophyllales. Following the divergence of *Physena*, gene duplication events near the base of core Caryophyllales gave rise to three clades within the CYP76AD1 lineage, here termed CYP76AD1-α, CYP76AD1-β and CYP76AD1-γ. All three clades are strongly supported with 100, 80 and 100% bootstrap support (BS), respectively. The CYP76AD1-α contains both CYP76AD1 genes that are functionally implicated in the betalain synthesis pathway (as mapped in Fig. 3a). The CYP76AD1-α, CYP76AD1-β, and CYP76AD1-γ exhibit asymmetric patterns of gene silencing and loss, with betalainic taxa typically possessing orthologs for all three loci but anthocyanic taxa possessing only one (Figs S3, S4). CYP76AD1-β and CYP76AD1-γ were present in a mutually exclusive fashion in six of the 15 anthocyanic transcriptomes. For example, in the anthocyanic Molluginaceae, CYP76AD1-γ was recovered in *Mollugo pentaphylla* and *M. verticillata*, whereas CYP76AD1-β was recovered in *M. cerviana*. CYP76AD1-β was also recovered in three Caryophyllaceae transcriptomes (Figs S1, S2). CYP76AD1-α was not detected in any anthocyanic transcriptomes (Figs 3a, S1, S2). In support of these results, BLAT and BLAST searches of the genomic scaffolds of the anthocyanic *Dianthus caryophyllus* (Caryophyllaceae) (Yagi *et al*., 2014) recovered the CYP76AD1-β isoform but were unable to detect the CYP76AD1-α isoform.

The DODA gene lineage underwent a minimum of 11 duplication events specific to the core Caryophyllales (Fig. 3b). The first deep duplication event occurred before the divergence of *Microtea*, generating two major clades within the Caryophyllales, here termed DODA-α and DODA-β. Both clades were weakly supported with 64% and 69% BS, respectively. DODA-α contains all three characterized DODA orthologs (i.e. from *B. vulgaris*, *M. jalapa* and *P. grandiflora*) that have been functionally implicated in the betalain synthesis pathway (as mapped in Fig. 3b). As with the CYP76AD1 lineage, the DODA-α and DODA-β clades also exhibited asymmetric patterns of gene silencing and loss, such that the DODA-α lineage has undergone a minimum of nine separate gene duplication events, but only two duplication events were inferred in the DODA-β lineage (Fig. 3b). Whereas isoforms of DODA-β were present in all 15 anthocyanic Caryophyllales transcriptomes, the DODA-α isoform was detected in only one, in *Spergularia media* (Caryophyllaceae); that is, this species possessed both isoforms of DODA (Figs S3, S4). BLAT and BLAST searches of the genomic scaffolds of the anthocyanic *D. caryophyllus* recovered the DODA-β isoform but were unable to detect the DODA-α isoform (Yagi *et al*., 2014).

**Fig. 4:**
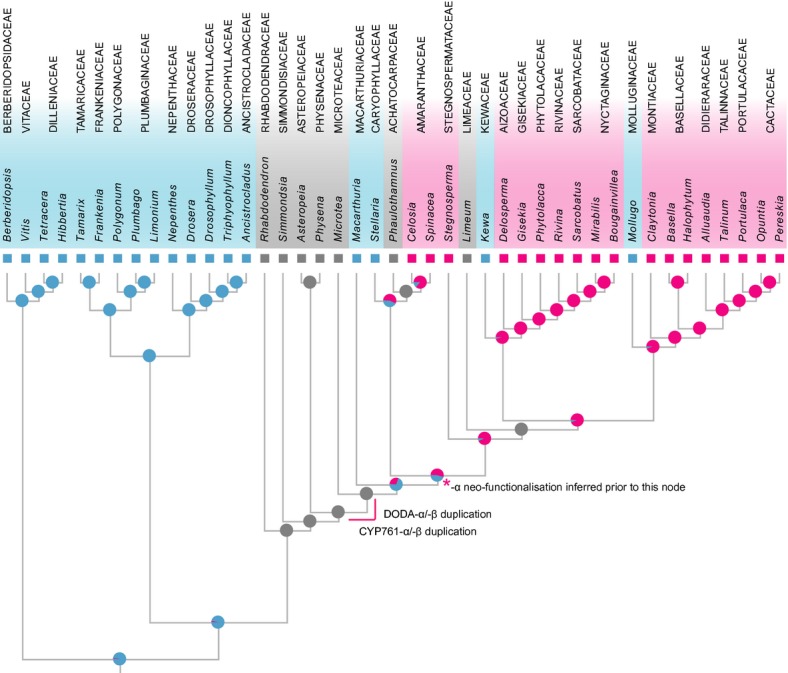
Topology derived from a maximum-likelihood (ML) analysis of a concatenated *matK/rbcL* dataset, with terminals coded blue for anthocyanin, pink for betalain and grey for missing data. Results of the ML reconstruction of dominant pigment type are also depicted; probabilities for character states at internal nodes are reported as pie charts. The solid pink line indicates the approximate phylogenetic location of the duplication event in the CYP76AD1 lineage that gave rise to CYP76AD1-α, -β, and -γ and the duplication event in the 4,5-dioxygenase (DODA) lineage that gave rise to DODA-α and β. The pink asterisk marks the node at which we infer neofunctionalisation of the CYP76AD1-α and DODA-α must have taken place on the basis of duplication and subsequent losses in the CYP76AD1 and DODA lineages.

In the *B. vulgaris* genome (Dohm *et al*., 2014) CYP76AD1-β and CYP76AD1-γ are located on chromosomes 1 and 9, respectively, whereas the betalain-specific CYP76AD1-α is located on chromosome 2 (Fig. 4). The *B. vulgaris* DODA-β locus is located on chromosome 4, as are four of the DODA-α homologs (data not shown). The remaining DODA-α homolog (Bv2_030650_ptjc.t1), which is functionally implicated in betalain pigmentation, resides on chromosome 2 and is located *c*. 50 kb from the betalain-specific CYP76AD1-α homolog (Bv2_030670_ucyh.t1). These two genes are separated by a single functionally uncharacterized locus (Bv2_030660_wpyx.t1) (Fig. 3c).

Likelihood-based ancestral state reconstructions of pigment evolution under the Mk1 model recovered anthocyanins as the most likely dominant pigment at the node uniting *Macarthuria* and remaining core Caryophyllales (betalain: 0.29, anthocyanin: 0.71; Fig. 4), but the probability of betalain pigmentation being dominant was higher at the following node, uniting Caryophyllaceae and remaining core Caryophyllales (betalain: 0.53, anthocyanin: 0.47). Three reversions to anthocyanic pigmentation were inferred, in the ancestors of Kewaceae, Molluginaceae and Caryophyllaceae (Fig. 4), although the node uniting Caryophyllaceae with *Phaulothamnus* and Amaranthaceae s.l. had approximately equal probabilioties of betalain (0.55) and anthocyanin (0.45) pigmentation. The inferred phylogenetic positions of the key duplications leading to the DODA-α/β, and CYP76AD1-α/β lineages are mapped onto the topology in Fig. 4.

**Fig. 5:**
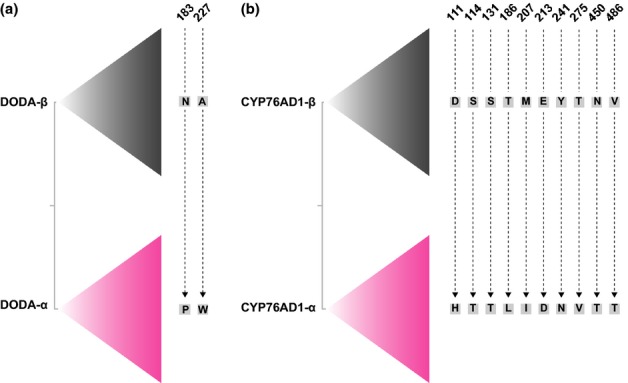
Diagnostic invariant residues (denoted by grey squares with black text) that distinguish the (a) 4,5-dioxygenase (DODA)-α and (b) CYP76AD1-α lineages, from their sister -β lineages. Denoted amino acid residues are invariant in the respective clades. CYP76AD1 residues are numbered on the basis of *Beta vulgaris* CYP76AD1-α and DODA residues are numbered on the basis of *Beta vulgaris* DODA-α.

Manual inspection of the DODA alignment identified two residues that are diagnostic for the DODA-α clade, including proline at site 183 and trytophan at site 227 (Fig. 5a; residues are numbered on the basis of *B. vulgaris* DODA-α). Positive selection analyses implemented in PAML revealed three additional sites (82, 102 and 115) that are under positive selection (*P* = 0.99). These three sites were variable among the in-paralog clades within the DODA-α lineage. For CYP76AD1, several residues were also found to be diagnostic for the CYP76AD1-α clade, including histidine at site 111, threonine at sites 114 and 131, leucine at site 186, isoleucine at site 207, aspartic acid at site 241 and valine at site 275 (Fig. 5b; residues are numbered on the basis of *B. vulgaris* CYP76AD1-α). The positive selection analyses implemented in PAML identified sites 111, 114, 131, 186, 241 and 275 as being under positive selection (*P* = 0.99) in CYP76AD1-α.

## Discussion

Our analyses demonstrate that the key betalain synthesis genes CYP76AD1 and DODA arose via gene duplications that are specific to the core Caryophyllales. Furthermore, the gene lineages containing CYP76AD1 and DODA have been subject to numerous and successive gene duplication events in core Caryophyllales. Nonetheless, within these broad Caryophyllales-specific radiations, all DODA and CYP76AD1 homologs that have been experimentally verified as functionally involved in the betalain synthesis pathway fall into single clades of related orthologs, despite being functionally characterized in phylogenetically diverse taxa. For example *MjCYP76AD2* (characterized in *Mirabilis jalapa*), *BvCYP76AD1* (characterized in *Beta vulgaris*), and *AcCYP76AD3* (characterized in *Amaranthus cruentus*) fall into the CYP76AD1-α clade, and all experimentally verified, functionally annotated DODA genes (*MjDODA* from *M. jalapa*, *PgDODA* from *P. grandiflora*, and *BvDODA* from *B. vulgaris*) fall into the DODA-α clade. Both the CYP76AD1-α and DODA-α clades arose via gene duplication events just before the divergence of *Microtea*, after which our reconstruction analyses suggest the probability of betalain pigmentation increases (Fig. 4; Brockington *et al*., 2011). The timing of the duplication events close to the inferred origin of betalain pigmentation, together with the observation that the CYP76AD1-α and DODA-α clades harbor all homologs functionally implicated in the betalain pathway, is significant. These observations imply that gene duplication and subsequent neofunctionalization were key events in the evolutionary origin of betalain pigmentation. However, we recognize that full confirmation of betalain-specific neofunctionalization will require future analysis of betalain-associated enzymatic activity between CYP76AD1-α and DODA-α and their sister clades, within single species.

Asymmetric patterns of gene loss in the CYP76AD1-α and DODA-α clades relative to their paralogous sister clades are informative with respect to the evolution of betalain-specific gene function. In contrast to the CYP76AD1-β and CYP76AD1-γ clades, no homologs of the CYP76AD1-α clade were identified in transcriptomes from anthocyanic taxa. We confirmed the absence of the CYP76AD1-α homolog from the genome of the anthocyanic *Dianthus caryophyllus*, suggesting that gene loss may underlie the general absence of CYP6AD1-α homologs from at least some anthocyanic transcriptomes. Exactly the same patterns of asymmetric gene loss occur in DODA-α vs DODA-β. Isoforms of DODA-β are present in 15 anthocyanic transcriptomes plus the genome of *D. caryophyllus*, but the DODA-α variant was only detectable from the transcriptome of a single putatively anthocyanic species, *Spergularia media*. The DODA-α sequence of *S. media* occupies the typical phylogenetic position of Caryophyllaceae, suggesting that this locus has been inherited and retained in *S. media* rather than acquired via horizontal gene transfer (HGT). Loss or downregulation of these loci in taxa with anthocyanin pigmentation also strongly implies that these clades have a betalain-specific function. Furthermore, it indicates that the betalain-specific function of the CYP76AD1-α and DODA-α homologs arose before the origin of these anthocyanic lineages. Consequently, we infer that the common ancestors to both the Caryophyllaceae and Molluginaceae each had a functional betalain synthesis pathway, and that the betalain-specific genes were then lost in the anthocyanic taxa because betalain pigmentation is no longer maintained. Loss of both the CYP76AD1-α and DODA-α isoforms is confirmed in only one anthocyanic species with a fully sequenced genome, *D. caryophyllus*. The bulk of our data were derived from transcriptomes and we therefore cannot distinguish between gene loss and downregulation. Although we appreciate that absence from the transcriptomes is not proof of absence, the common expression of the DODA-β in 15 different anthocyanic transcriptomes compared with the presence of the DODA-α in a single anthocyanic transcriptome is compelling.

The asymmetric loss or downregulation of the DODA-α and CYP76AD1-α homologs in anthocyanic taxa suggests an interesting mechanistic difference underlying the mutual exclusion of these two pigment types. Whereas the suppression of anthocyanin pigmentation in betalain taxa appears to be achieved at the regulatory level (Shimada *et al*., 2004, 2005, 2007; Hatlestad *et al*., 2014), the long-term exclusion of betalain pigments from anthocyanic taxa is likely mediated through gene loss. These patterns of gene loss or downregulation in betalain-pigmented taxa also inform our understanding of the homoplastic distribution of anthocyanic lineages and betalain-pigmented clades. Given the intercalation of anthocyanic and betalain pigmented lineages, previous analyses concluded that derived instances of anthocyanin pigmentation are reversals from an ancestral betalain condition. However, in a previous study we showed that the phylogenetic disintegration of the polyphyletic Molluginaceae s.l. broadens the distribution of anthocyanin pigmentation such that each major clade of betalain taxa is now subtended at, or towards its base by an anthocyanic lineage (Brockington *et al*., 2011). The anthocyanic Molluginaceae s.s. are sister to the betalain-pigmented Portulacineae, the anthocyanic *Kewa* (formerly included in *Hypertelis*; Christenhusz *et al*., 2014) are sister to the betalain-pigmented Aizoaceae, Gisekiaceae, Phytolaccaceae and Nyctaginaceae, and the anthocyanic Caryophyllaceae are sister to the betalain-pigmented Amaranthaceae s.l. We previously argued that this pattern was consistent with multiple origins of betalain pigmentation from an ancestral anthocyanic condition (Brockington *et al*., 2011). However, the loss or downregulation of CYP76AD1-α and DODA-α homologs from anthocyanic lineages argues against multiple origins of betalain pigmentation, and instead suggests that a betalain-pigment pathway was fully functioning in the common ancestor to the Caryophyllaceae, Molluginaceae and *Kewa*. A single origin of betalain pigmentation seems more likely in light of these data.

The phylogenetic timing and location of the duplication events giving rise to CYP76AD1-α and DODA-α do not coincide with the previously identified hotspots of gene duplication within Caryophyllales that are putatively associated with whole genome duplication events (Dohm *et al*., 2012, 2014; Yang *et al*., 2015). We therefore reasoned that the DODA and CYP76AD1 genes might be located physically close together and thus would be subject to more localized duplication events. When we examined the physical location of the *B. vulgaris* DODA and CYP76AD1 homologs we found that the betalain-specific DODA-α homolog (Bv2_030650_ptjc.t1) and betalain-specific CYP76AD1-α homolog (Bv2_030670_ucyh.t1) are in close proximity on the same scaffold of chromosome 2 in the *B. vulgaris* genome. The fact that these genes are < 50 kb apart greatly increases the probability of both loci being subject to the same localized segmental duplication(s), which may explain the phylogenetic proximity of the duplication events that led to the betalain-specific DODA-α and CYP76AD1-α clades. Alternatively, this clustering of functionally related but nonhomologous loci is suggestive of an operon-like cluster, albeit of small gene number, such as has been described for other plant secondary metabolic pathways including triterpenes, steroidal and isoquinoline alkaloids, and cyanogenic glycosides (Boycheva *et al*., 2014). A MYB gene (the R locus) that regulates both DODA-α and CYP76AD1-α in *B. vulgaris* (Hatlestad *et al*., 2014), is also located on chromosome 2 and is linked to the CYP76AD1-α (Y) locus (Keller, 1936; Goldman & Austin, 2000), further supporting the concept of a metabolic operon.

We explored the role for neofunctionalization in the evolution of the betalain-specific DODA-α and CYP76AD1-α loci by examining diagnostic molecular substitutions and patterns of selection. Christinet *et al*. (2004) proposed that paralogous copies of DODA possess diagnostic residues that are positioned close to the putative catalytic site of the DODA protein. In our taxon-dense dataset, two of these residues hold up as invariant sites that distinguish DODA-α and DODA-β. Immediately following the histidine at site 182, which is universally conserved across all DODA genes (Christinet *et al*., 2004), a proline at site 183 is invariantly present in the DODA-α clade but is almost invariantly asparagine and never proline in the DODA-β clade. Similarly, at site 227 a tryptophan is invariably present in DODA-α whereas alanine is invariably present at the same site in DODA-β. Analyses of selection patterns in the DODA lineage indicate that DODA-α underwent positive selection following the duplication event that gave rise to DODA-α and its sister lineage DODA-β. Furthermore, positive selection analyses revealed a number of variable sites that are under positive selection (*P* = 0.99) within DODA-α, including sites 82, 102 and 115. Interestingly, the residues at these sites sometimes vary between paralogous clades within DODA-α suggesting possible adaptive evolution after duplication events within the DODA-α lineage.

Analyses of selection patterns also indicate that the CYP76AD1-α lineage underwent positive selection following the duplication event that gave rise to CYP76AD1-α and its sister lineage CYP76AD1-β, at sites 111, 114, 131, 186, 241 and 275. However, these putatively functional significant changes are spread across the CYP76AD1 protein and are hence less obviously associated with one active site. These results are in line with analyses of mammalian cytochrome P450 proteins, which have documented that substrate recognition is mediated by several substrate recognition sites (SRSs) that are broadly distributed across the protein (Gotoh, 1992). Point mutations have been shown to significantly affect substrate recognition (Gotoh, 1992). It is likely that the betalain-specific function of CYP76AD1-α arose in a switch of substrate specificity towards l-DOPA, and hence these identified residues are an important starting point in understanding the evolution of a putative specificity change.

Comparison of the asymmetric radiation in DODA-α and DODA-β is intriguing. Only two gene duplications are inferred (one within Nyctaginaceae, one within Portulacaceae) in DODA-β, whereas a minimum of nine duplications are inferred at the familial level or above in DODA-α, including four at the base of the clade containing Aizoaceae, Nyctaginaceae, Phytolaccaceae and Sarcobataceae, three within the Portulacineae, and three within the Amaranthaceae. These duplications largely coincide with hotspots of gene duplication that are likely the product of genome duplication (Yang *et al*., 2015). Therefore, we suggest that the asymmetric radiation of DODA-α genes is the result of differential retention of DODA-α homologs vs DODA-β, rather than the product of localized segmental duplications of chromosomal regions that contain the DODA-α homologs. It is tempting to speculate that this radiation of DODA-α homologs is connected with the radiation of the betalain color tool-kit and that different paralogs of DODA-α may contribute to differential coloring in some way. With this in mind, it is interesting that the paralogous clades within DODA-α differ in diagnostic residues at sites that are suggested to be under positive selection, suggesting adaptive evolution following gene duplication within the DODA-α clade. Alternatively these paralogs could be differentially expressed and enable finer control of differential coloring patterns through the development of betalain-pigmented taxa. Functional analysis of these additional paralogs will ultimately be necessary to determine their *in planta* significance.

### Conclusion

In summary, we propose that the betalain synthesis pathway arose in part through coincidental duplication events in the DODA and CYP76AD1 lineages. We infer that these duplication events gave rise to the betalain-specific DODA-α and CYP76AD1-α clades. Several lines of evidence suggest that this betalain-specific neofunctionalization occurred in the DODA-α and CYP76AD1-α clades, including: the origin of these clades just before the probable origin of betalain pigmentation; the asymmetric loss or downregulation of DODA-α and CYP76AD1-α homologs in anthocyanic lineages; the restricted distribution of isoforms functionally implicated in betalain synthesis to the DODA-α and CYP76AD1-α clades; and the asymmetric radiation of the DODA-α lineage in betalain taxa vs the low copy number of DODA-β detectable from transcriptomes in equivalent species. These patterns imply that betalain pigmentation arose once, early in the evolution of the Caryophyllales, and then was lost in anthocyanic lineages. Furthermore our data imply that the long-term exclusion of betalain pigments from anthocyanic taxa is likely mediated through gene loss, rather than at the regulatory level, as is the case with the suppression of anthocyanin in betalain species. We identify numerous diagnostic residues and sites under positive selection that are good candidates with which to explore the evolution of this specificity change in these putatively neofunctionalized clades. Finally we identify that the functionally related but otherwise nonhomologous DODA-α and CYP76AD1-α genes are physically located close together in the *B. vulgaris* genome, suggestive of a possible operon cluster in relation to the betalain synthesis pathway. Future experiments should aim to assess the betalain-specific activity of the CYP76AD1-α and DODA-α lineages relative to their paralogous sister clades, explore the functional significance of the asymmetric radiation in the DODA-α lineages, and seek to understand how specific amino acid changes have contributed to the evolution of betalain-specific activity. Finally, understanding the evolutionary forces that led to multiple losses of betalain pigmentation and reversions to the anthocyanic condition remains a key unanswered question.

## Supporting Information

**Fig. S1** Taxon-labeled phylogeny of the CYP76AD1 lineages (and CYP76AD1–γ). **Fig. S2** Taxon-labeled phylogeny of the CYP76AD1 lineages (CYP76AD1-α, CYP76AD1-β). **Fig. S3** Taxon-labeled phylogeny of the 4,5-dioxygenase (DODA) lineages (DODA-β). **Fig. S4** Taxon-labeled phylogeny of the 4,5-dioxygenase (DODA) lineage (DODA-α).

**Table S1** Sources of the transcriptome data

**Table S2** Information for 14 newly sequenced transcriptomes: provenance, herbarium vouchers, type of material

**Table S3** List of 4,5-dioxygenase (DODA) gene accessions and corresponding taxa

**Table S4** List of CYP76AD1 gene accessions and corresponding taxa
