# Supplementary material for: Lineage-specific gene radiations underlie the evolution of novel betalain pigmentation in Caryophyllales
Source: New Phytol. 2015 May 13;207(4):1170–80. doi: 10.1111/nph.13441 (PMC4557044; doi:10.1111/nph.13441)
Supplement: Supplementary file 1 [file nph0207-1170-sd1.pdf]

***New Phytologist* Supporting Information Figs S1–S4**

Article title: Lineage-specific gene radiations underlie the evolution of novel betalain pigmentation In Caryophyllales

Authors: Samuel F. Brockington, Ya Yang, Fernando Gandia-Herrero, Sarah Covshoff, Julian M. Hibberd, Rowan Sage, Gane K. S. Wong, Michael J. Moore and Stephen A. Smith

Article acceptance date: 03 April 2015

The following Supporting Information is available for this article:

Fig. S1 Taxon-labeled phylogeny of the CYP76AD1 lineages (and CYP76AD1–γ).

Fig. S2 Taxon-labeled phylogeny of the CYP76AD1 lineages (CYP76AD1-α, CYP76AD1-β)

Fig. S3 Taxon-labeled phylogeny of the 4,5-dioxygenase (DODA) lineages (DODA-β).

Fig. S4 Taxon-labeled phylogeny of the 4,5-dioxygenase (DODA) lineage (DODA-α).

Table S1 Sources of the transcriptome data (separate Excel file)

Table S2 Information for 14 newly sequenced transcriptomes: provenance, herbarium vouchers, type of material (separate Excel file)

Table S3 List of 4,5-dioxygenase (DODA) gene accessions and corresponding taxa (separate Excel file)

Table S4 List of CYP76AD1 gene accessions and corresponding taxa (separate Excel file)

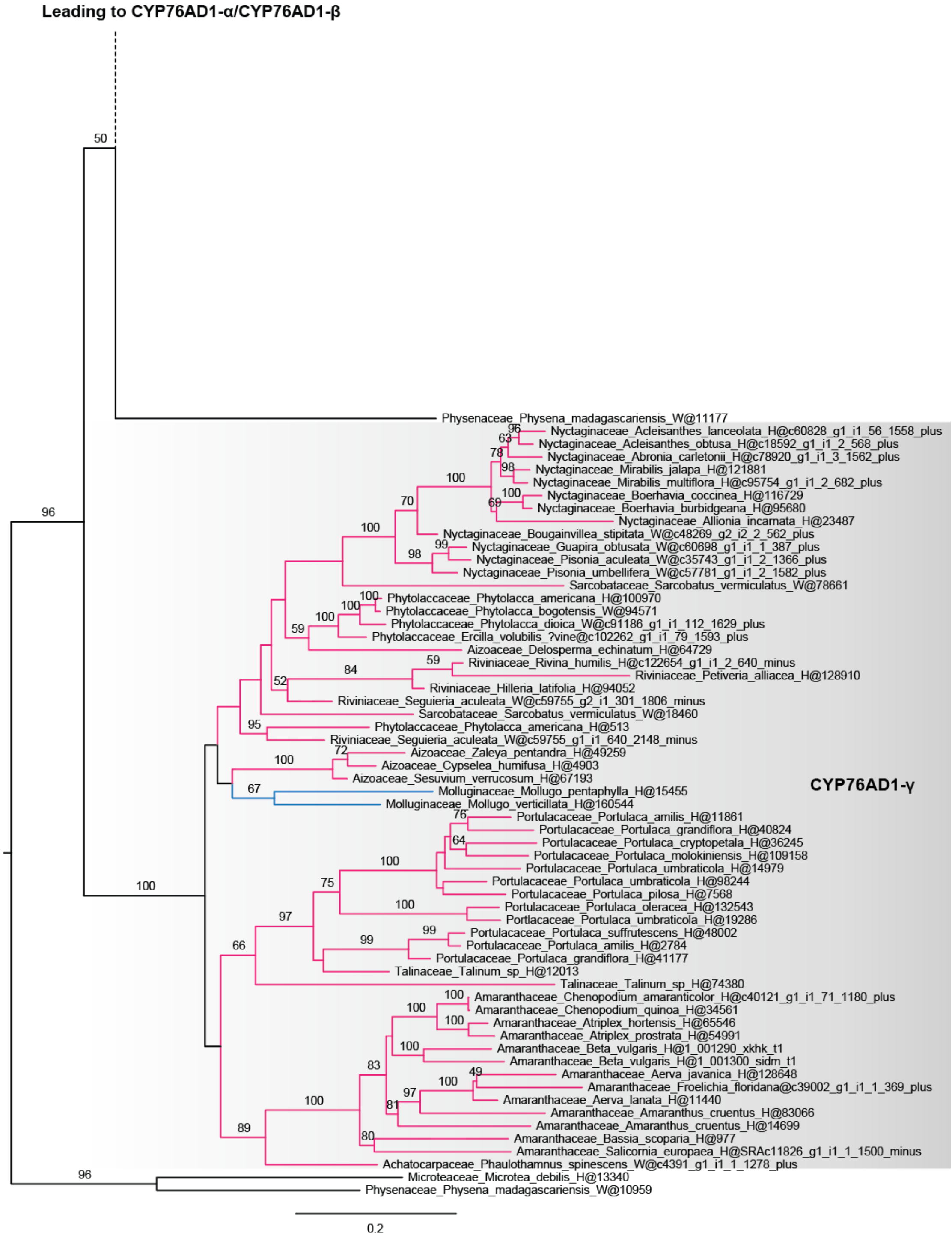

Fig. S1 Taxon-labeled phylogeny of the CYP76AD1 lineages (CYP76AD1–γ).

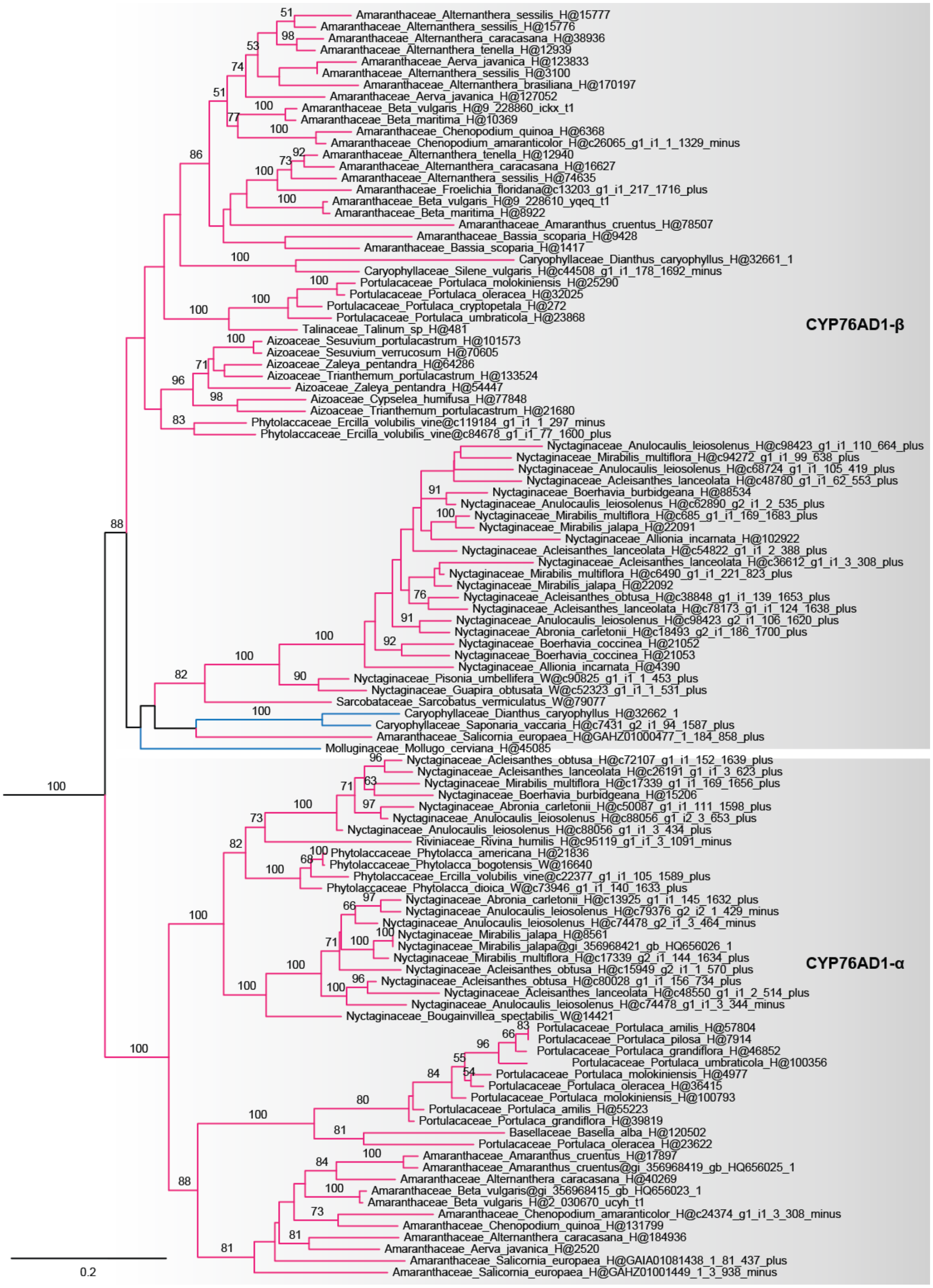

Fig. S2 Taxon-labeled phylogeny of the CYP76AD1 lineages (CYP76AD1-α, CYP76AD1-β).

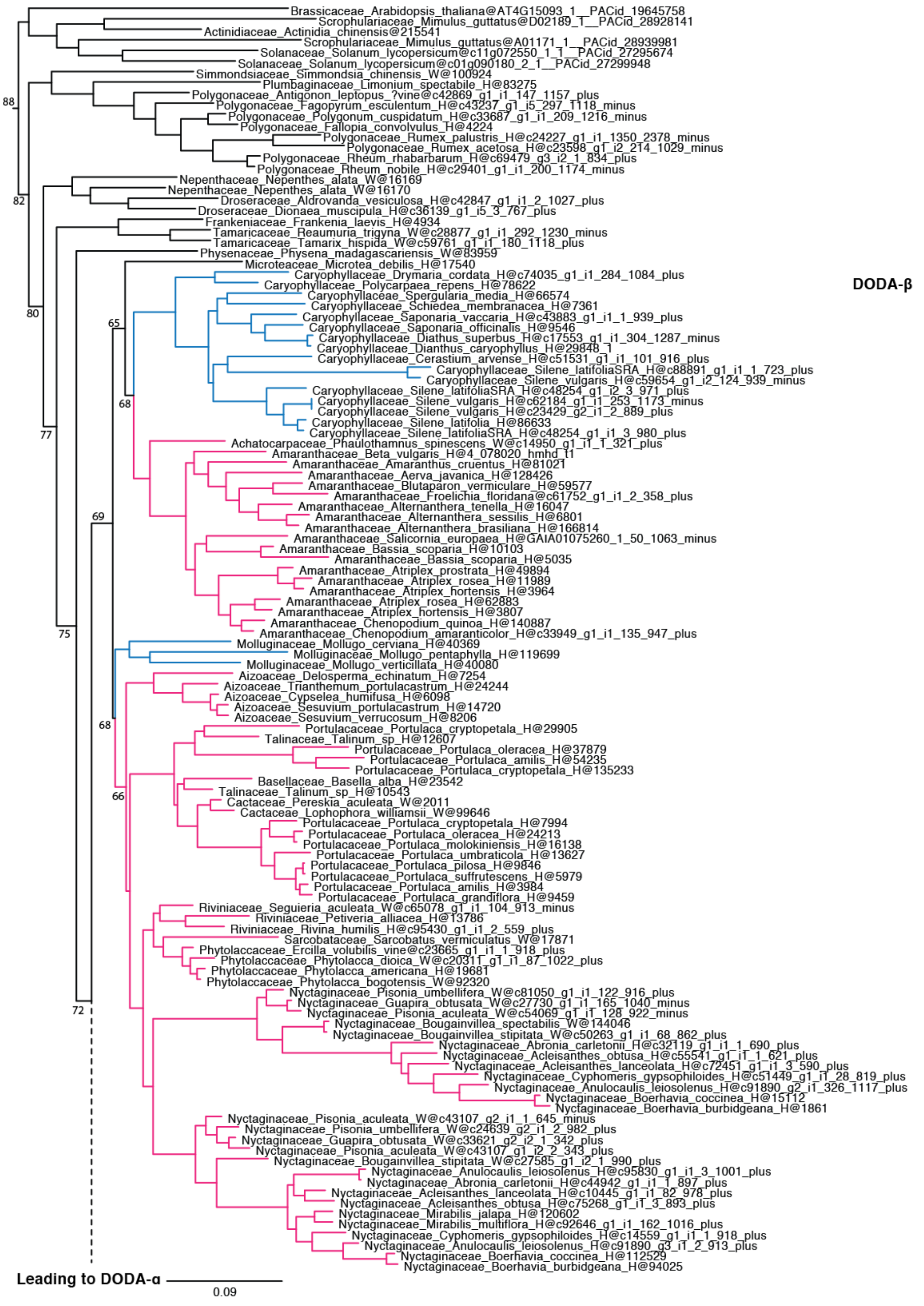

Fig. S3 Taxon-labeled phylogeny of the 4,5-dioxygenase (DODA) lineages (DODA-β).

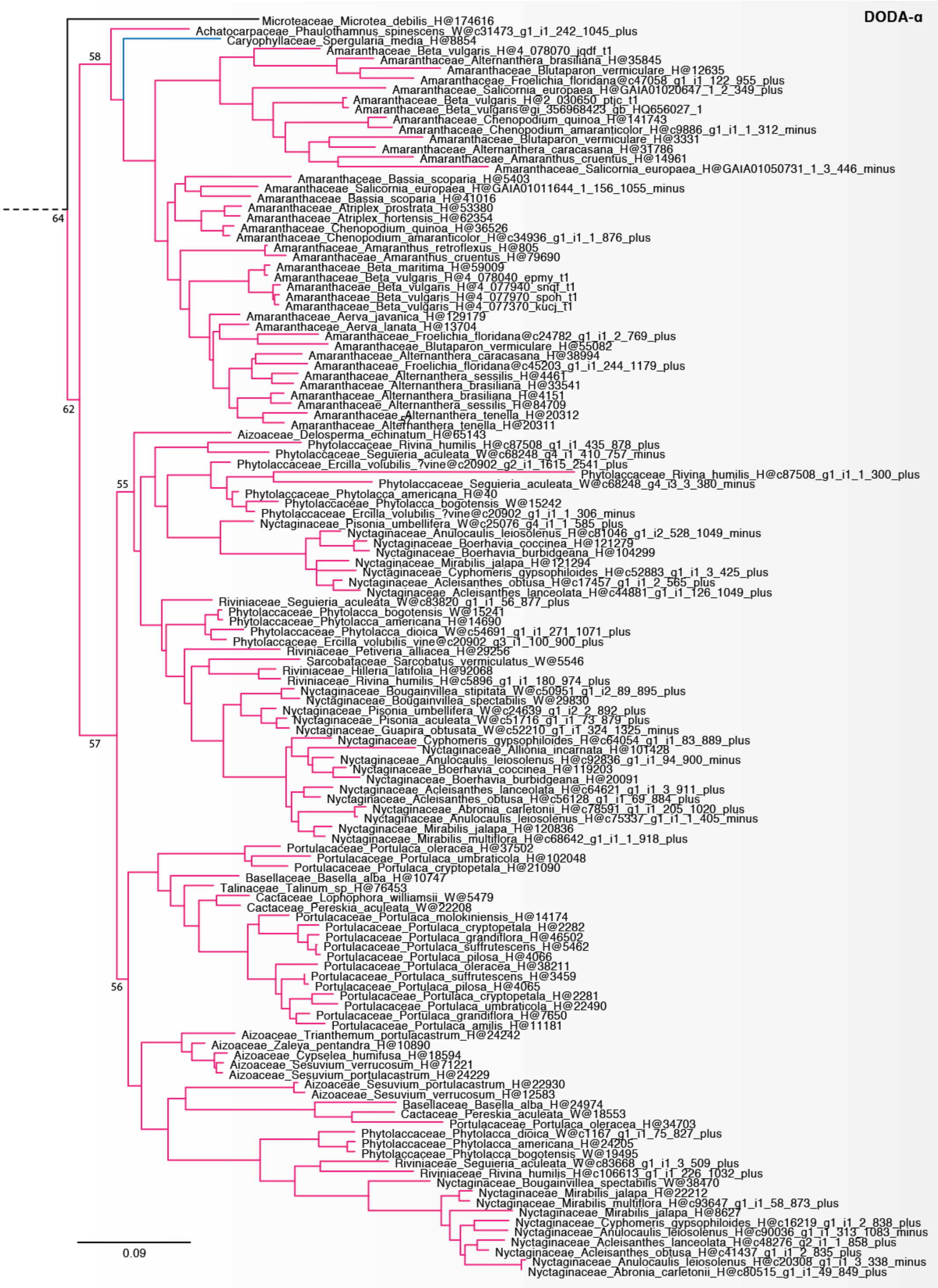

Fig. S4 Taxon-labeled phylogeny of the 4,5-dioxygenase (DODA) lineage (DODA-α).
